# Integrating Spatial Multi-Omics and Machine Learning to Unravel the Role of PANoptosis in Bladder Cancer Prognosis and Immunotherapy Response

**DOI:** 10.32604/or.2025.064331

**Published:** 2025-08-28

**Authors:** Liangju Peng, Tingting Cai, Peihang Xu, Cong Chen, Qingzhi Xiang, Yiping Zhu, Dingwei Ye, Yijun Shen

**Affiliations:** 1 Department of Urology, Fudan University Shanghai Cancer Center, Shanghai, 200032, China; 2 Department of Oncology, Shanghai Medical College, Fudan University, Shanghai, 200032, China; 3 Department of Nursing, Fudan University Shanghai Cancer Center, Shanghai, 201321, China

**Keywords:** PANoptosis, bladder cancer, spatial transcriptome, tumor microenvironment, immunotherapy

## Abstract

**Background:** Studies have reported the special value of PANoptosis in cancer, but there is no study on the prognostic and therapeutic effects of PANoptosis in bladder cancer (BLCA). This study aimed to explore the role of PANoptosis in BLCA heterogeneity and its impact on clinical outcomes and immunotherapy response while establishing a robust prognostic model based on PANoptosis-related features. **Methods:** Gene expression profiles and clinical data were collected from public databases. Spatial heterogeneity of cell death pathways in BLCA was evaluated. Consensus clustering was performed based on identified PANoptosis genes. Cell death pathway scores, molecular, and pathway activation differences between different groups were compared. Protein-protein interaction (PPI) network construction was constructed, and immune-related gene sets, tumor immune dysfunction and exclusion (TIDE) scores, and SubMap analysis were used to evaluate immunomodulator expression and immunotherapy efficacy. Ten machine learning algorithms were utilized to develop the most accurate predictive risk model, and a nomogram was created for clinical application. **Results:** BLCA demonstrated a spatially heterogeneous distribution of pyroptosis, apoptosis, and necroptosis. Notably, T effector cells significantly colocalized with total apoptosis. Two PANoptosis modes were identified: high PANoptosis (high. PANO) and low PANoptosis (low. PANO). High. PANO was associated with worse clinical outcomes and advanced tumor stage, and increased activation of immune-related and cell death pathways. It also showed increased infiltration of immune cells, elevated expression of immunomodulatory factors, and enhanced responsiveness to the immunotherapy. The PANoptosis-related machine learning prognostic signature (PMLS) exhibited strong predictive power for outcomes in BLCA. CSPG4 was identified as a key gene underlying prognostic and therapeutic differences. **Conclusion:** PANoptosis shapes distinct prognostic and immunological phenotypes in BLCA. PMLS offers a reliable prognostic tool. CSPG4 may represent a potential therapeutic target in PANoptosis-driven BLCA.

## 1 Introduction

Bladder cancer (BLCA) is a common urological malignancy characterized by various pathological subtypes, with urothelial carcinoma (transitional cell carcinoma) being the most prevalent, accounting for over 90% of cases. Less common subtypes include squamous cell carcinoma, adenocarcinoma, and small cell carcinoma [1,2]. Traditionally, bladder cancer has been considered resistant to conventional radiotherapy and chemotherapy, leading to surgery-based treatment strategies such as transurethral resection, partial cystectomy, or radical cystectomy combined with chemotherapy or immunotherapy [3,4]. The emergence of immune checkpoint inhibitors, which target the immunosuppressive tumor microenvironment, has significantly improved bladder cancer treatment. However, only a subset of patients benefits from durable responses, underscoring the importance of identifying predictive biomarkers for treatment optimization [5–7].

Programmed cell death (PCD) functions as a physiological mechanism initiated by the organism to maintain internal homeostasis. PCD appears in various forms, such as apoptosis, pyroptosis, and necroptosis, each extensively studied [8]. Traditionally seen as separate pathways, recent evidence suggests intricate interactions among apoptosis, pyroptosis, and necroptosis. Apoptosis is a genetically regulated form of PCD, along with distinct morphological changes and is regulated by caspase activation through intrinsic or extrinsic pathways [9]. Pyroptosis is a form of PCD marked by the disruption of plasma membrane integrity, driven by the activation of inflammasome sensors [10]. Necroptosis, a genetically regulated form of lytic cell death, is primarily triggered by the activation of receptor-interacting protein kinase 1 (RIPK1) and receptor-interacting protein kinase 3 (RIPK3) [11]. A novel form of PCD, known as PANoptosis, is a distinct form of inflammatory cell death resulting from the interplay between pyroptosis [12], apoptosis and necroptosis, mediated by the assembly of PANoptosome complexes that incorporate elements from these cell death pathways [13]. Recent studies have shown that apoptosis, necroptosis, and pyroptosis are interconnected at multiple levels, with the formation of the PANoptosome complex and the subsequent activation of PANoptosis [14]. In times of cellular upheaval or infection, sensory proteins recognize changes, leading to PANoptosome formation and eventual PANoptosis activation [15]. PANoptosis is typically generated by cytoplasmic pattern recognition receptors (PRRs) upon sensing diverse stimuli, including pathogens, pathogen-associated molecular patterns (PAMPs), damage-associated molecular patterns (DAMPs), or cytokines. These molecular assemblies are composed of inflammasomes, caspase-8, and additional molecules, driving cell death [16]. Research identifies ZBP1, PIPK1, AIM2, and NLRP12 as triggers for PANoptosome assembly upon detecting specific signals, leading to PANoptosis [17]. When cancer cells block PCD pathways, PANoptosis offers a distinct pathway to eliminate malignant cells and impede tumor advancement [18,19]. Thus, PANoptosis is not merely a parallel coexistence of three distinct cell death pathways but rather a network capable of triggering or amplifying multiple cell death signals under specific conditions. Compared to studying a single form of cell death, PANoptosis provides a more comprehensive reflection of the complex and interwoven cell death network between tumor cells and the immune microenvironment [20]. The PANoptosis concept presents a new approach to combat tumors, with recent studies emphasizing its significance in certain cancer types.

Various studies have emphasized the crucial role of broad-spectrum cell death in cancerous growths. For example, within liver cancer, different forms of widespread cell death categorize the illness into three subgroups. Of these, individuals with increased levels of genes linked to cell death have a bleak projection, reduced survival rates, and amplified invasion of immune cells [21]. In cases of head and neck tumors, a high score for widespread cell death may suggest spread to lymph nodes, advancement to later clinical stages, resistance to chemotherapy and avoidance of the immune system [22]. In renal tumors, Xu and colleagues formulated a signature for broad-spectrum cell death founded on genes related to this process (BAX, CASP1, CASP8, PYCARD) and found that a high score for broad-spectrum cell death was linked to heightened infiltration of the immune system, increased effectiveness of immunotherapy, and the detection of a novel target for treatment, PYCARD [23]. BLCA shares certain immunological characteristics with the aforementioned tumors [24,25], such as high tumor heterogeneity and partial sensitivity to immunotherapy, suggesting that similar phenomena may also exist in BLCA. The study’s preliminary bioinformatics analysis revealed that certain genes associated with apoptosis or pyroptosis, such as CSPG4, were also significantly correlated with necroptosis-related genes and inflammatory factors. This suggests that multiple cell death pathways may be simultaneously activated in BLCA, supporting the hypothesis of a coordinated ‘multi-pathway’ PANoptosis mechanism. Besides, the successful application of immune checkpoint inhibitors in BLCA, coupled with the influence of cell death pathways on immune cell infiltration and antigen presentation, highlights PANoptosis as a potential key regulator of the response to immunotherapy [26–28]. Nevertheless, the influence of PANoptosis on the prognosis and treatment in BLCA patients remains unstudied. BLCA patients were grouped according to the PANoptosis gene in this research. This study aimed to analyze variations in prognosis and treatment among different patient cohorts, as well as investigate variances in molecular activation and pathway activation across these groups. To create an optimal prognostic risk model, a combination of 10 machine learning algorithms was utilized to construct a nomogram.

## 2 Materials and Methods

### 2.1 Data Acquisition and Initial Processing

**Figure A1:**
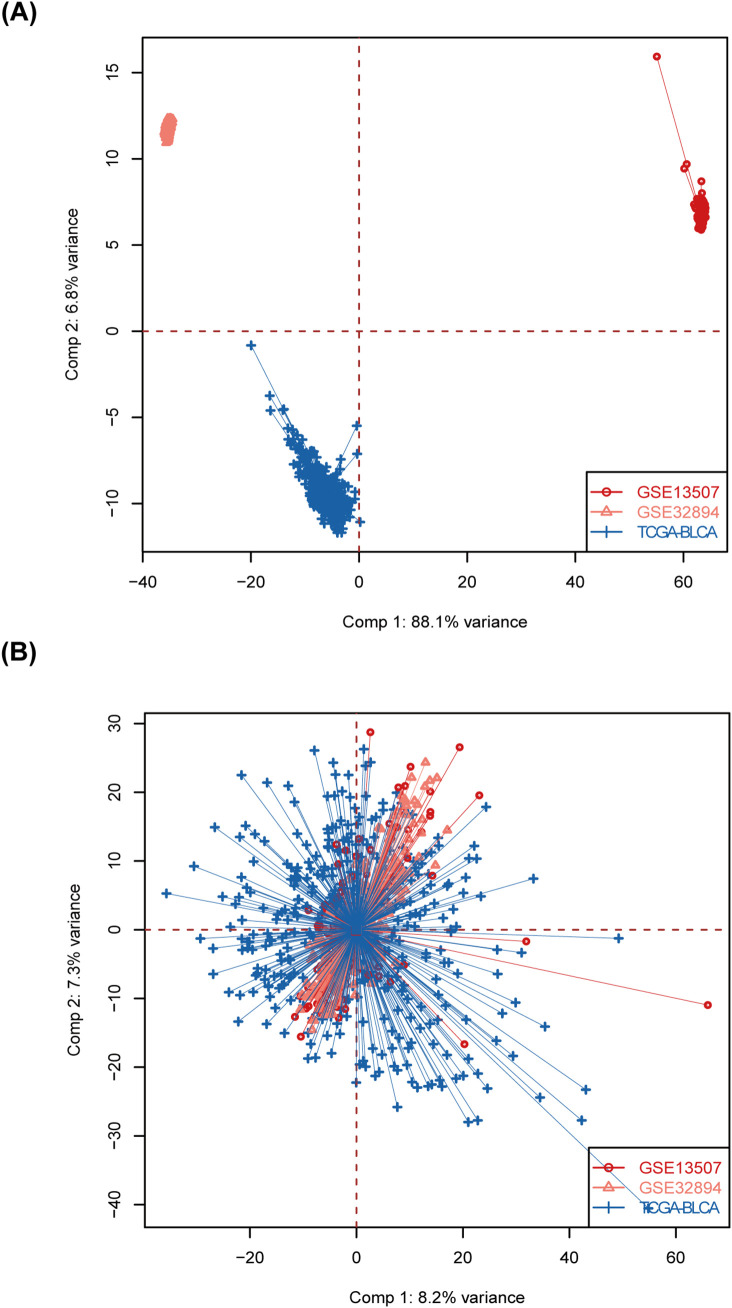
Batch effects removal. **(A)** Raw PCA for combined expression profile. **(B)** Combat PCA for combined expression profile

Gene expression profiles and clinical information were downloaded from publicly available platforms, which included The Cancer Genome Atlas (TCGA, https://www.cancer.gov/ccg/research/genome-sequencing/tcga (accessed on 16 May 2025)) and Gene Expression Omnibus (GEO, https://www.ncbi.nlm.nih.gov/geo/ (accessed on 16 May 2025)). The study encompassed three groups, consisting of a total of 871 samples: TCGA-BLCA (*n* = 409), GSE32894 (*n* = 224), GSE13507 (*n* = 165), and GSE48075 (*n* = 73). The R package ‘TCGAbiolinks’ (V.2.26.0) was used for data acquisition of TCGA-BLCA. All gene expression profiles were uniformly normalized to transcripts per million (TPM) units. To enhance data reliability, messenger RNAs were discarded when the TPM value was under 1 in more than 90% of specimens. Patients without corresponding mRNA profiles, clinical data, or follow-up details were excluded to minimize potential biases. To make the results more comparable, we reduced the non-biological difference using the ‘sva’ (V.3.46.0) package (Appendix Fig. A1). GSE32894 and GSE13507 were considered as representative of treatment-naive bladder cancer samples, while the TCGA-BLCA dataset indeed includes a more detailed treatment background considered as treated samples. The study mainly referred to previous studies on core genes related to PANoptosis [29], as well as key gene sets from databases such as Hallmark (https://www.gsea-msigdb.org/gsea/msigdb (accessed on 17 May 2025)), Kyoto Encyclopedia of Genes and Genomes (KEGG, https://www.kegg.jp/), and Reactome (https://reactome.org/) related to pyroptosis, necroptosis, and apoptosis. The study preliminarily screened and integrated these genes to form a candidate gene set. Download somatic mutation profiles from the cBioPortal database (https://www.cbioportal.org/), excluding non-coding variants. Filter out silent mutations and non-coding RNA mutations based on whole-exome sequencing coverage, and calculate the Tumor Mutational Burden (TMB) value using the specified equation (TMB (mutations/Mb) = mutation number/30 Mb).

### 2.2 Spatial Analysis

Spatial transcriptome data was obtained from GEO database (GSE171351), including four tumor samples (Bladder1204, Bladder8, Bladder72, and Bladder371) [30]. This study utilized ‘Scanpy’ (V.1.11.0) to process spatial data. The original Spatial RNA sequencing data underwent quality control, which included the removal of low-quality cells and genes. After the preprocessing stage, the gene expression values are normalized using the ‘scanpy.pp.normalize_total()’ function with the default parameter ‘target_sum=1e4’, which scales the total counts per cell to 10,000, thereby facilitating direct comparisons between cells. Subsequently, the normalized data undergoes log transformation using ‘scanpy.pp.log1p()’ to stabilize variance and mitigate the influence of highly expressed genes. Following this, gene set scoring is conducted using ‘scanpy.tl.score_genes()’, based on predefined features associated with ECROPTOSIS, PYROPTOSIS, APOPTOSIS, and PANoptosis, which enables the quantification of pathway activity at the single-cell level. Additionally, to investigate the performance of different cell populations in T cell toxicity, the study applied a T cell toxicity gene set comprising known genes associated with T cell toxicity (GZMB, GZMH, GZMK, GZMA, TIA1, PRF1, LAMP1, GNLY, FASLG, SLAMF7, ZAP70, CD69, TNF). Specifically, genes within this gene set were first selected and weighted based on their expression levels, after which the scores were normalized to eliminate potential biases between cells by comparing each cell’s score with its spatial distribution. The study also spatially mapped the idle data, integrating the gene set scores with the actual spatial positions of the cells within the tissue. The scanpy.pl.spatial() function was employed to generate a map illustrating the spatial positions and scores of the cells, thereby demonstrating the spatial heterogeneity of the scores.

### 2.3 Consensus Cluster Analysis

**Figure A2:**
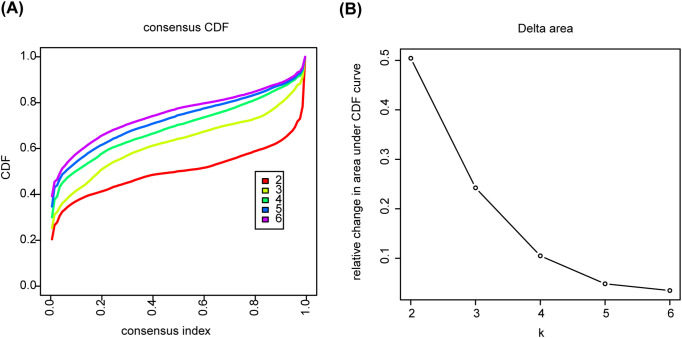
Determination of optimal cluster number using consensus clustering. **(A)** The consensus cumulative distribution function (CDF) plot. **(B)** Delta area plot under CDF curve

In the TCGA-BLCA cohort, BLCA patients were grouped by four specific chemokine genes through the utilization of the ‘ConsensusClusterPlus’ (V.1.62.0) R package. The clustering utilized 50 iterations to ensure stability and mitigate random initialization effects. The study evaluated the ideal cluster count by assessing the cumulative distribution function (CDF) curve for values of k = 2 to k = 6. The variable k represents the predefined number of clusters that the algorithm utilizes to partition the samples. Each value of k corresponds to a potential number of distinct molecular subtypes among the patients. Based on the consensus CDF plot and Delta area tracking, k = 2 was selected as the optimal number of clusters, which exhibited the most stable clustering result with significant changes in the Delta area under the CDF curve (Appendix Fig. A2).

### 2.4 Gene Set Variation Analysis (GSVA) Analysis, Enrichment Analysis and Protein-Protein Interaction (PPI) Network Establishment

Through the implementation of GSVA analysis, we assessed the scores of NECROPTOSIS, PYROPTOSIS, APOPTOSIS, and PANoptosis in various clusters. The ‘limma’ (V.3.54.2) package was utilized to detect Differentially expressed genes (DEGs), with an adjusted *p*-value threshold of less than 1. Following this, the analysis was carried out using the ‘ClusterProfiler’ (V.4.6.2) tool, using Gene Ontology (GO) and KEGG databases. To visualize the protein relationships, the STRING (https://string-db.org/) database was used to construct PPI networks for the DEGs, shedding light on the intricate interactions between proteins involved in diverse biological processes [31]. Additionally, the study included 50 traditional oncology gene sets from the Hallmark collection, drawing from existing biological insights, and performed Gene Set Enrichment Analysis (GSEA) analysis on all samples using the R package ‘clusterProfiler’ (version 4.0.5). The metrics of Normalized enrichment score (NES) and false discovery rate (FDR) were utilized as measures of enrichment, with gene sets displaying |NES| > 1 and FDR < 0.25 considered significantly enriched. The visualization of the findings was accomplished with the assistance of the R package ‘enrichplot’ (version 1.12.1).

### 2.5 Comparison of Immunomodulator Compilation and Immunotherapy Efficacy

Expression of mRNA involved in immunomodulation was analyzed, alongside the frequencies of methylation, amplification, and gene deletion across various subtypes of BLCA. Comprehensive details are documented in a previously published study [32]. Furthermore, the study obtained 33 sets of immune-related genes and utilized the GSVA algorithm to calculate scores that represent the degree of immune cell infiltration within the tumors [33]. The study then retrieved the tumor immune dysfunction and exclusion (TIDE) scores for bladder cancer cases from the TIDE website (https://tide.dfci.harvard.edu/), which served to evaluate the immune evasion and the potential benefits of immunotherapy. In addition, the study employed the SubMap module in GenePattern (https://cloud.genepattern.org/gp/pages/index.jsf (accessed on 15 May 2025).) to forecast the sensitivity to the immune checkpoint inhibitor therapy.

### 2.6 Immunohistochemistry (IHC) Staining

The expression of CSPG4 was estimated using IHC of patients with BLCA. The primary antibody was a rabbit monoclonal anti-CSPG4 (anti-CSPG4 antibody: Cat. A24955, ABclonal, Wuhan, China) used at a dilution of 1:1000. The formalin-fixed paraffin-embedded (FFPE) sections underwent deparaffinization, rehydration, and were treated with 3% hydrogen peroxide at 25°C for 12 min. Antigen retrieval was conducted using a 0.01 mol/L citrate buffer (pH 6.0) heated to 95°C to 98°C for 25 min. Following this, the slides were allowed to cool for 1 h before being blocked with 5% bovine serum albumin (BSA, Cat. P0252, Beyotime, Shanghai, China) in PBS (phosphate buffered saline) for 30 min at room temperature. Staining with the primary antibody took place overnight at 4°C. The next day, incubation of the sections was performed with the secondary antibody. The secondary antibody was a HRP-conjugated Goat antibody (anti-Rabbit IgG (H+L) antibody: Cat. AS014, ABclonal, Wuhan, China) utilized at a dilution of 1:10,000. Following this tissue sections were stained with a diaminobenzidine (DAB) kit (Cat. DA1010, Solarbio, Beijing, China) for 5 min. Counterstaining was performed with hematoxylin (BSA, Cat. C0107, Beyotime, Shanghai, China) for 30 s, then washed with running water for 5 min. Finally, the slides underwent tissue differentiation, dehydration, transparency and sealing sheet. The paraffin specimens were obtained from 20 BLCA patients at our center.

This research received approval from the Ethics Committee of Fudan University Shanghai Cancer Center (No. 050432-4-2307E), following the Declaration of Helsinki, and written informed consent was obtained from each patient.

### 2.7 Combination of 10 Machine Learning Algorithms to Build a Novel Risk Model

After filtering DEGs in bladder cancer, the study then developed a reliable PANoptosis-related machine learning prognostic signature (PMLS) with a comprehensive machine learning analysis procedure, including 10 machine learning methods, and the details could be found in a prior published literature [34]. All of the models were ordered as average values of the C-index among TCGA-BLCA, GSE32894, and GSE13507 cohorts. Remove combinations with too low C-index or overfitting. Finally, PMLS was calculated by $\text{PMLS }=\sum_{k=1}^{n}\left(coef.i*expression.i\right)$.

### 2.8 Nomogram Establishment

Combining multivariate Cox regression analysis and prior published articles, we employed covariates including sex, histological grade, histologic type, tumor stage, age, tumor status, and PMLS to establish a novel nomogram, where tumor stage was annotated according to the American Joint Committee on Cancer (AJCC) 7th edition [35]. To determine the nomogram’s predictive capability and accuracy, Kaplan-Meier (K-M), receiver operating characteristic (ROC) and calibration analysis were performed. To evaluate the calibration performance of the nomogram, the Hosmer-Lemeshow test was calculated, and *p* > 0.05 indicates favorable fitness between predicted probabilities and observed outcomes.

### 2.9 Regulon Analysis

The package ‘RTN 2.12.1’ was used to create a transcriptional regulatory network (regulon) comprising 23 ‘regulatory’ genes linked to induction/repression targets. An analysis of mutual information and Spearman’s rank correlation was applied to infer potential connections among regulators and all possible targets in the transcriptome profile. Permutation analysis (*n* = 1000) was carried out to eliminate associations with FDR > 0.00001. An approach of bootstrapping was applied to remove unstable correlations through a thousand resamples, achieving a consensus bootstrapping rate exceeding 95%. Data processing included filtering out weaker associations in triangles involving two regulators and common targets via inequality filtering, while estimating individual regulator activity using two-sided GSEA [36].

### 2.10 Statistical Analysis

R software (version 4.2.2) and Python (version 3.9) were utilized for statistical analysis. Kruskal-Wallis and Wilcoxon tests were utilized to compare group differences. *p* < 0.05 was set to be statistically significant.

## 3 Results

### 3.1 Spatial Heterogeneity of Cell Death Pathways in BLCA and Correlation with Progression

**Figure 1:**
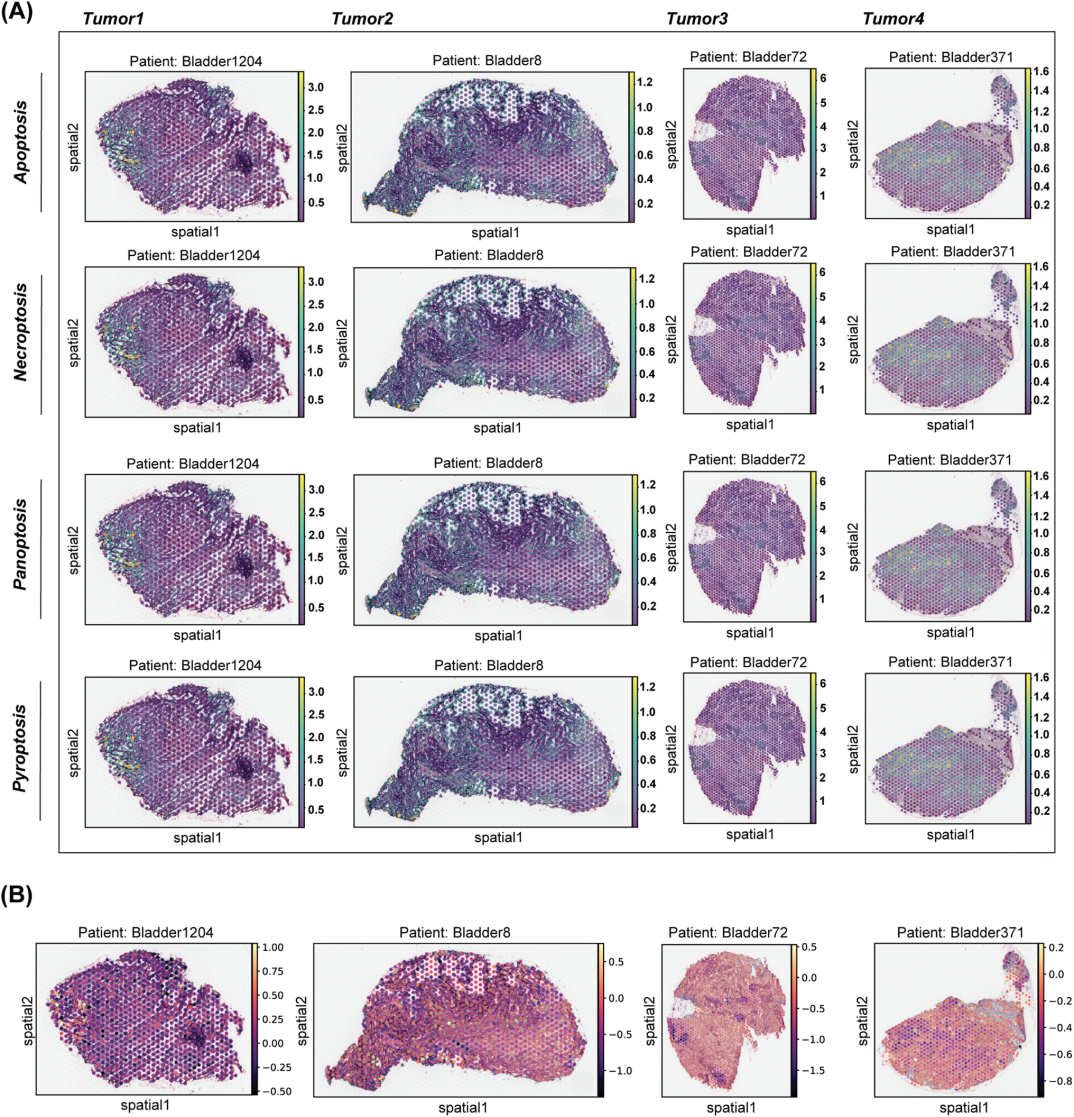
Distribution of NECROPTOSIS, PYROPTOSIS, APOPTOSIS, PANoptosis and T cytotoxicity scores in tissues. **(A)** This study examines the spatial expression of various modes of cell death in tumor samples from different patients. A spatial map illustrates the distribution of each cell death pattern within the tumor areas of patients Bladder1204, Bladder8, Bladder72, and Bladder371. **(B)** Spatial plot showed the distribution of four cell death modes across four patients

**Figure A3:**
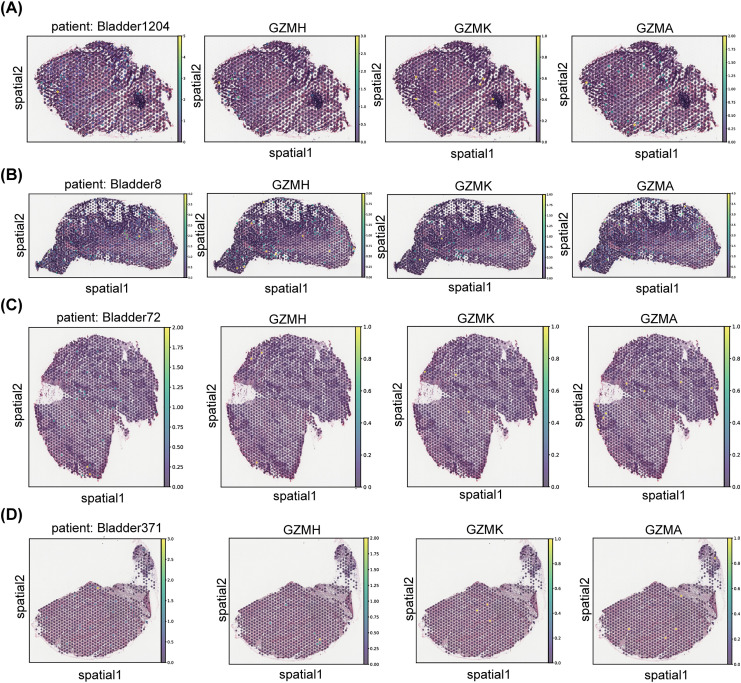
The spatial distribution of gene expression for CD8^+^ T effector molecules, specifically various granzyme family members, in bladder cancer samples is presented. Panels (A–D) illustrate the spatial expression patterns of GZMH, GZMK, GZMA, and GZMB in tumor sections from different bladder cancer patients (Bladder1204, Bladder8, Bladder72, and Bladder371, respectively)

Based on spatial transcriptome analysis, the study found that bladder cancer exhibits distinct spatial distributions of cell death patterns, particularly in pathways such as pyroptosis, apoptosis, necroptosis, and PANoptosis. These various cell death patterns demonstrate significant spatial heterogeneity across different tumor regions (Fig. 1A). Notably, tumor samples from different patients reveal that the intensities and distributions of these cell death patterns vary at different spatial locations, highlighting the diversity of programmed cell death within the tumor microenvironment and its potential influence on tumor progression. Furthermore, several modes of cell death display similar spatial distribution patterns. For instance, the distribution profiles of PANoptosis and PANoptosis are significantly comparable across multiple patient samples. This observation suggests an intrinsic connection between these death pathways, which may be triggered or regulated by common factors in the tumor microenvironment, such as the inflammatory response. Additionally, by analyzing the spatial distribution of T cell toxicity scores alongside each death mode, we observed a high correlation between the peak values of the T cell toxicity score and the peak distribution of panoptosis as well as T effector molecules, which included GZMH, GZMK and GZMA (Fig. 1B, Appendix Fig. A3). These results may indicate that PANoptosis, as a form of programmed cell death, plays a crucial role in tumor immune evasion, through the interplay with the immune system, potentially contributing to immunotherapy.

### 3.2 Identification of Two Patterns of PANoptosis in BLCA

**Figure 2:**
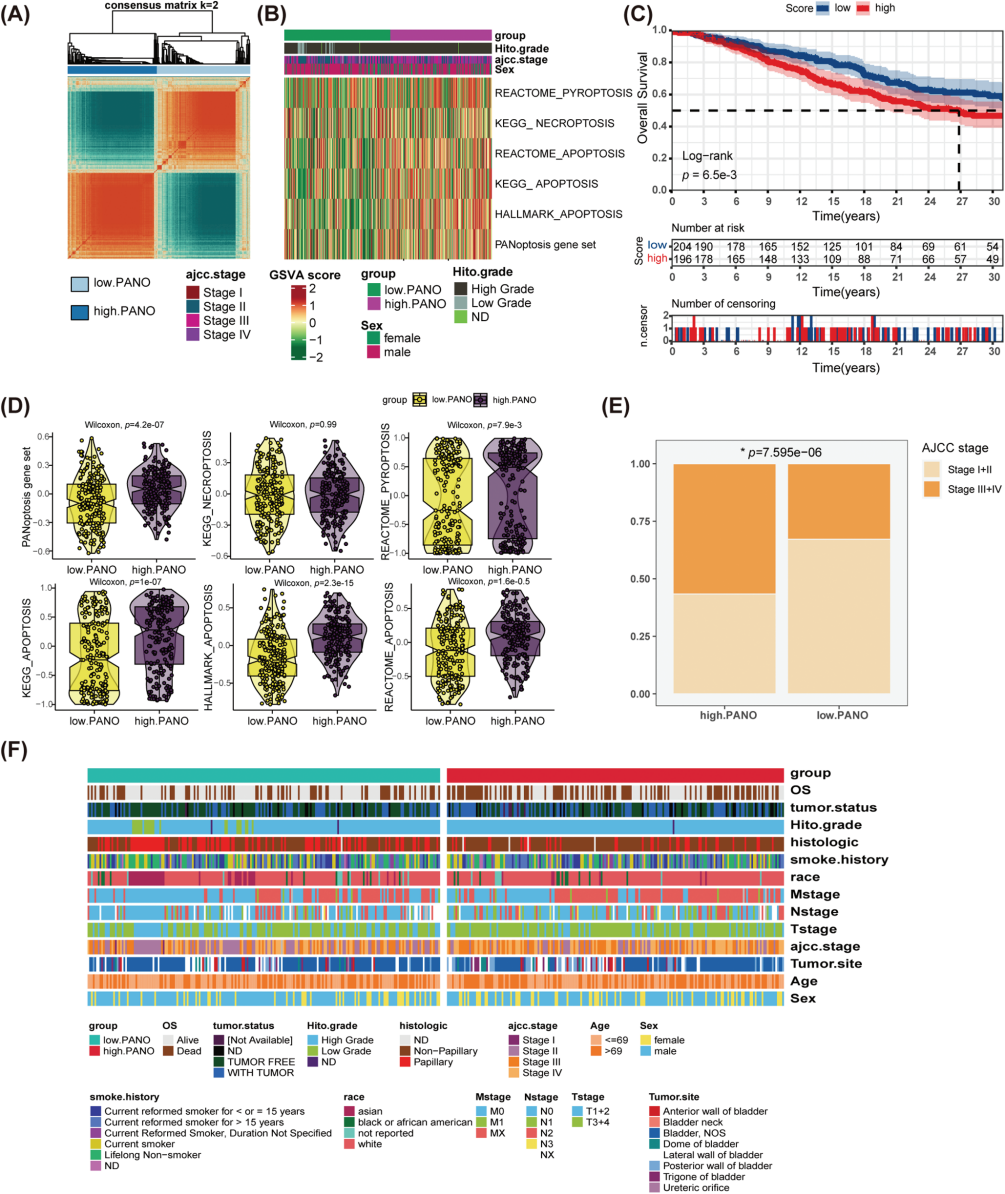
Identification of Two Distinct PANoptosis Patterns in bladder cancer (BLCA) and Their Clinical Implications. **(A)** Unsupervised clustering analysis on BLCA patients utilizing PANoptosis-related gene sets; **(B)** distinct patterns of programmed cell death (PCD) between low. PANO and high. PANO; **(C)** K-M plot; **(D)** comparison of three types of PCD; **(E)** comparison of tumor stage between the two subtypes, *p < 0.05; **(F)** distribution of clinicopathological features of the two subtypes

Utilizing an unsupervised clustering algorithm, the study identified two distinct patterns of PANoptosis in BLCA (Fig. 2A). To explore variations in cell death statuses within these subtypes, a total of six gene sets associated with various death processes, including PANoptosis, necroptosis, pyroptosis, and apoptosis, were examined. By employing GSVA, individual scores were computed for each patient, indicating that individuals in high. PANO exhibited notably elevated activation scores for the PANoptosis gene set (*p* = 4.2e−07), REACTOME_PYROPTOSIS (*p* = 7.9e−3), KEGG_APOPTOSIS (*p* = 1e−07), HALLMARK_APOPTOSIS (*p* = 2.3e−15), and REACTOME_APOPTOSIS (*p* = 1.6e−05) (Fig. 2B–D). Moreover, individuals in high. PANO demonstrated decreased overall survival rates (*p* = 6.5e−3, Fig. 2C) and exhibited a higher prevalence of high-stage tumors (*p* = 7.595e−06, Fig. 2E). Additionally, a thorough comparison of clinicopathological features between the two subtypes demonstrated that high. PANO displayed more indicators of unfavorable prognosis, such as an increased proportion of patients with metastasis and higher mortality rates (Fig. 2F). These results suggest that high. PANO signifies a subtype characterized by a bleak clinical prognosis and elevated PANoptosis score.

### 3.3 Validation in External Cohorts

**Figure 3:**
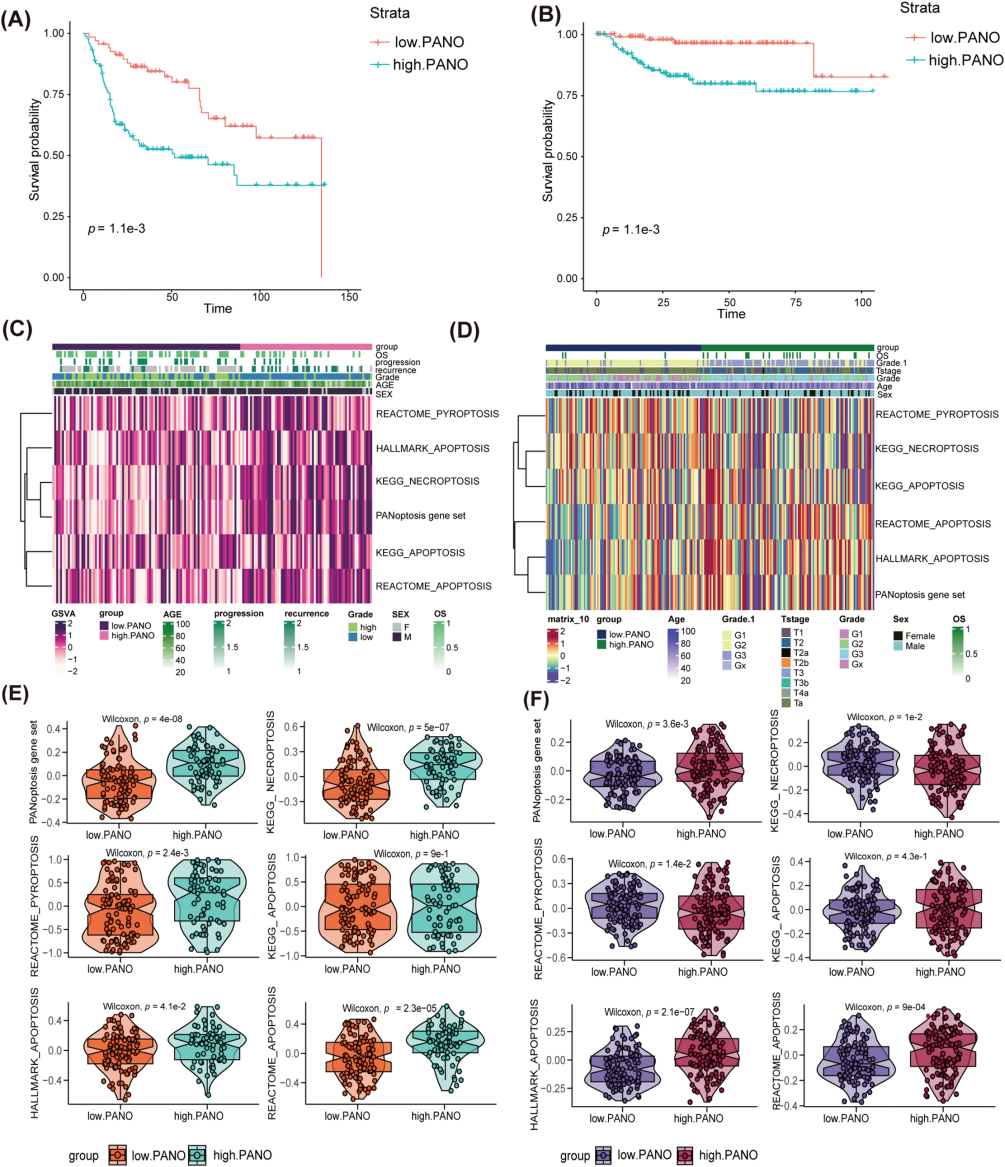
Validation in external cohorts. **(A)** K-M plot in GSE13507 cohort; **(B)** K-M plot in GSE32894 cohort; **(C)** distinct patterns of programmed cell death (PCD) between low. PANO and high. PANO in GSE13507 cohort; **(D)** distinct patterns of three types of PCD between low. PANO and high. PANO in GSE32894 cohort; **(E)** comparison of three types of PCD in GSE13507 cohort; **(F)** comparison of three types of PCD in GSE32894 cohort

**Figure A4:**
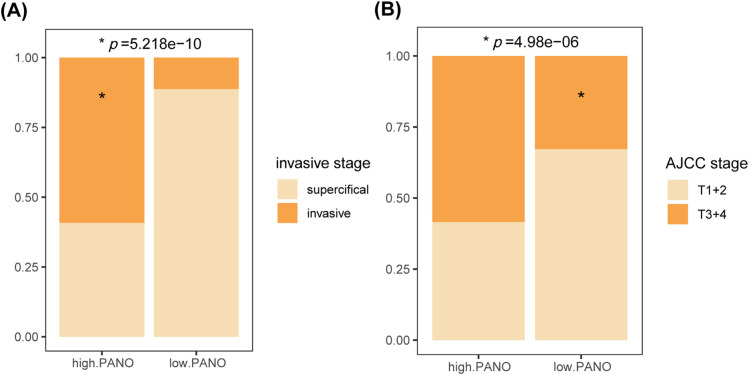
Validation in the external cohort. **(A)** Comparison of tumor invasive stage and **(B)** AJCC stage among the two subtypes in the GSE48075 cohort. *, *p* < 0.05

To validate the results obtained in the TCGA-BLCA cohort, the study conducted a similar analysis in three additional external cohorts. Both in GSE13507 (Fig. 3A) and GSE32894 cohorts (Fig. 3B), high. PANO subtype showed shorter overall survival (OS) compared to low. PANO subtype (all *p* < 0.05). Besides, high. PANO subtype exhibited a higher prevalence of invasive tumors (*p* = 5.218e−10) and higher stage tumors (*p* = 4.98e−06) in GSE48075 (Appendix Fig. A4). Additionally, high. PANO and low. PANO subtypes exhibited different status in PANoptosis, necroptosis, pyroptosis, and apoptosis in both cohorts (Fig. 3C,D). Notably, high. PANO displayed significantly elevated scores for the PANoptosis gene set (*p* = 4.2e−08), REACTOME_PYROPTOSIS (*p* = 2.4e−3), HALLMARK_APOPTOSIS (*p* = 0.04), KEGG_NECROPTOSIS (*p* = 5e−07), KEGG_APOPTOSIS (*p* = 0.90), and REACTOME_APOPTOSIS (*p* = 2.3e−05) in the GSE32894 cohort (Fig. 3E). In the GSE13507 cohort, high. PANO also showed high scores for the PANoptosis gene set (*p* = 3.6e−3), REACTOME_PYROPTOSIS (*p* = 0.01), HALLMARK_APOPTOSIS (*p* = 2.1e−07), KEGG_NECROPTOSIS (*p* = 0.01), KEGG_APOPTOSIS (*p* = 0.43), and REACTOME_APOPTOSIS (*p* = 9e−04) (Fig. 3F). Taken together, these results support the classification of the high. PANO subtype is correlated with an unfavorable prognosis.

### 3.4 High. PANO Subtype Presented More Immune-Related Pathway Activation

**Figure 4:**
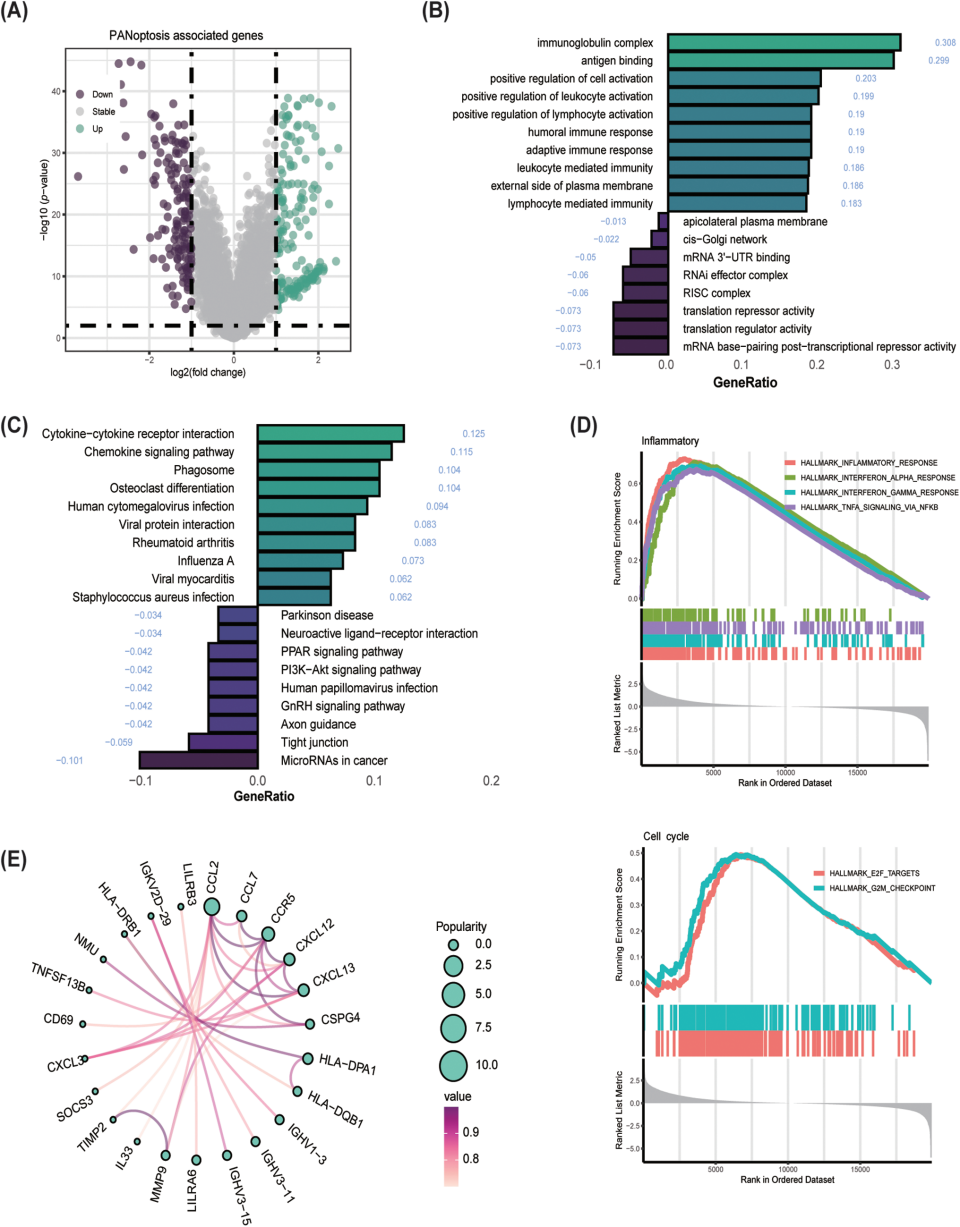
Enrichment analysis and protein-protein interaction (PPI) network establishment. **(A)** Volcano plot showed differential genes (DEGs) among the two groups; **(B)** GO enrichment analysis; **(C)** KEGG enrichment analysis; **(D)** GSEA analysis. **(E)** Establishment of PPI network

To conduct a deeper examination into the relationship between the two groups, the study identified and analyzed 218 DEGs (Fig. 4A) utilizing the GO and KEGG databases. Analysis using GO revealed that the high. PANO subtype exhibited increased activation of immune-related pathways (Fig. 4B). These pathways included the immunoglobulin complex, antigen binding, positive regulation of leukocyte activation, humoral and adaptive immune response, and leukocyte-mediated immunity. Contrastingly, the low. PANO subtype showed heightened activation of pathways associated with posttranscriptional and translational control. Notable pathways included the apicolateral plasma membrane, cis-Golgi network, mRNA 3^′^-UTR binding, and RNA-induced silencing complex (RISC). Additionally, KEGG (Fig. 4C) analysis indicated that patients classified as high. PANO exhibited elevated activation scores in cytokine-cytokine receptor interaction and chemokine signaling pathways, which implies increased inflammatory activity. Furthermore, several pathways linked to cell proliferation, development, and apoptosis were found to be enriched in low. PANO BLCA. GSEA analysis revealed higher levels of inflammatory activation in the high. PANO group (Fig. 4D). Furthermore, the activation of cell cycle-related pathways indicated a more proliferative capacity in this phenotype, which is also associated with a poorer prognosis and higher grade. A PPI network was generated to clarify protein-level interactions among DEGs. After removing unconnected proteins, 23 hub genes were identified (Fig. 4E), with the CCL2/CCR2 axis, CXCL12/CXCR7/CXCR4 axis, and CCL7 garnering significant attention. CCL2 and its receptor CCR2 promote tumor cell proliferation and metastasis by attracting monocytes and macrophages in the tumor microenvironment. Additionally, CCL2 also facilitates tumor immune escape by recruiting regulatory T cells (Tregs) and myeloid-derived suppressor cells (MDSCs) to inhibit anti-tumor immune [37]. CXCL12 and its receptors, CXCR4 and CXCR7, are commonly found in high levels in various cancers, including BLCA. CXCL12 stimulates tumor cell proliferation, movement, and invasion by activating of the AKT and ERK pathways. Furthermore, its interaction with fibroblasts within the tumor microenvironment facilitates immune evasion by tumor cells [38]. Similarly, elevated levels of CCL7 in the tumor microenvironment have been linked to the advancement and spread of different cancers. CCL7 boosts tumor invasiveness and aids in immune evasion by attracting a variety of immune cells, particularly macrophages [39].

### 3.5 High. PANO Is a Potential Immunotherapy-Responsive Phenotype

**Figure 5:**
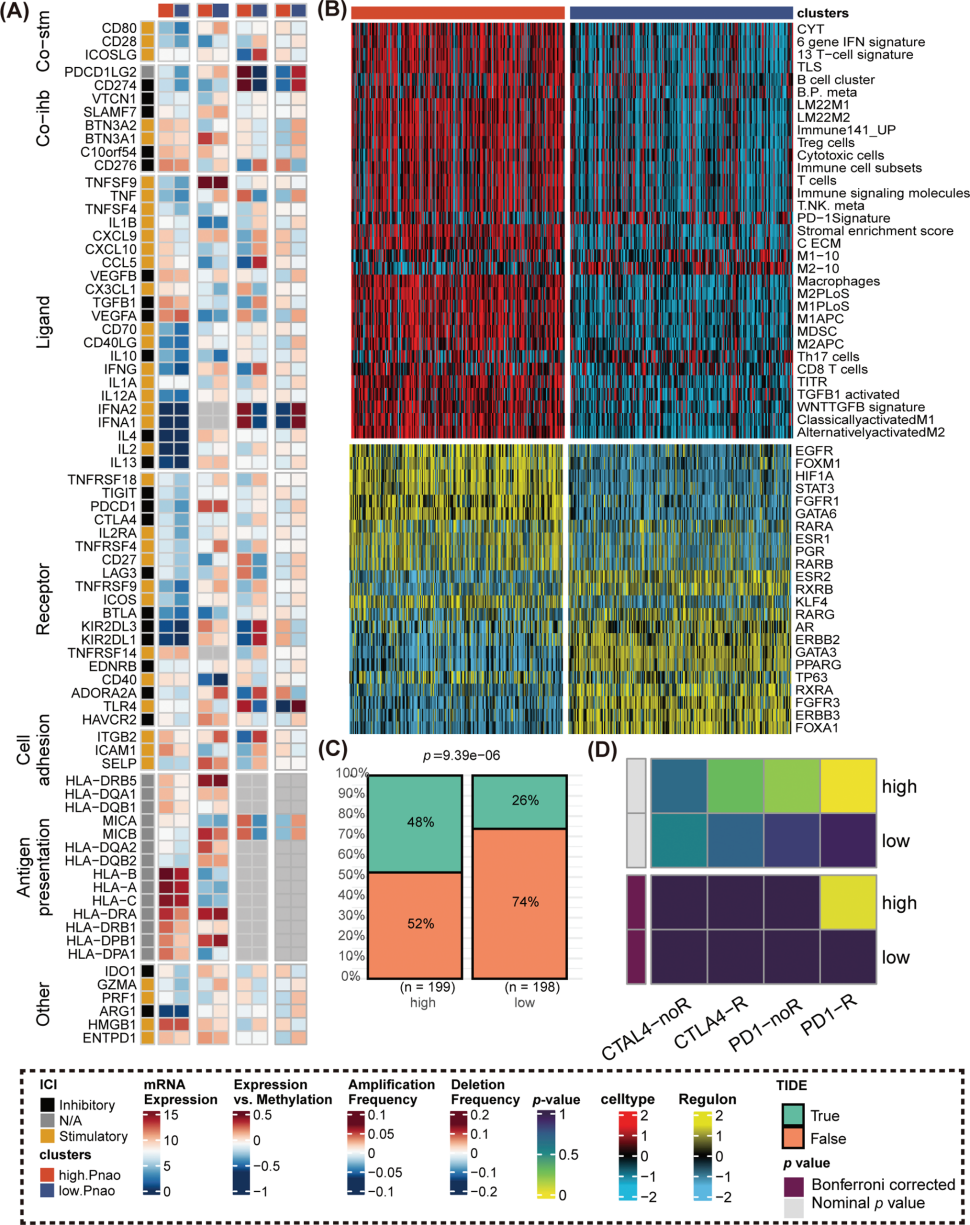
Comparison of immunomodulators, immune cell infiltrations, regulon level, and immunotherapy response. **(A)** Comparison of immunomodulators between high. PANO and low. PANO subtypes, involving mRNA expression, expression vs methylation, amplification and deletion frequency. **(B)** Distinct infiltrations of immune cells and expression level of regulons; **(C)** TIDE analysis. **(D)** Submap analysis.

The primary molecular differences between the two groups are primarily found in the chemokine axis, immune cell chemotaxis, and activation. Gene expression of immunomodulators varied between the subtypes (Fig. 5A), with CXCR10, CXCL9, and CCL5 showing higher expression in high. PANO patients compared to low. PANO [32]. Notably, CCL5 amplification frequency was higher in low. PANO, potentially contributing to progression and metastasis in BLCA through JAK2/STAT4 signaling activation. Higher methylation levels in low. PANO might lead to epigenetic silencing of CCL5, weakening its biological function [38]. Based on 33 immune-related gene sets, the study illustrated the various immune cell infiltrations. As shown in Fig. 5B, high. PANO subtype exhibited a more activated immune cell infiltration score. Prominently, elevated levels of a variety of transcription factors linked with tumor progression, such as EGFR, FOXM1, STAT3, FGFR1, and GATA6, were identified in the high PANO subtype [40–42]. Targeted inhibitors of these transcription factors hold promise in promoting the outcome of immunotherapy. This highlights the crucial role of immunotherapy in managing high. PANO patients. The utility of the TIDE method was confirmed to be high. PANO subtype contained more immunotherapy responders compared to the low. PANO (*p* = 9.39e−06, Fig. 5C). Consistently, SubMap analysis also manifested patients in high. PANO group was a potential responder to anti-PD−1 therapy (Fig. 5D).

### 3.6 Construction Prognostic Models Based on Multiple Machine Learning Strategies

**Figure 6:**
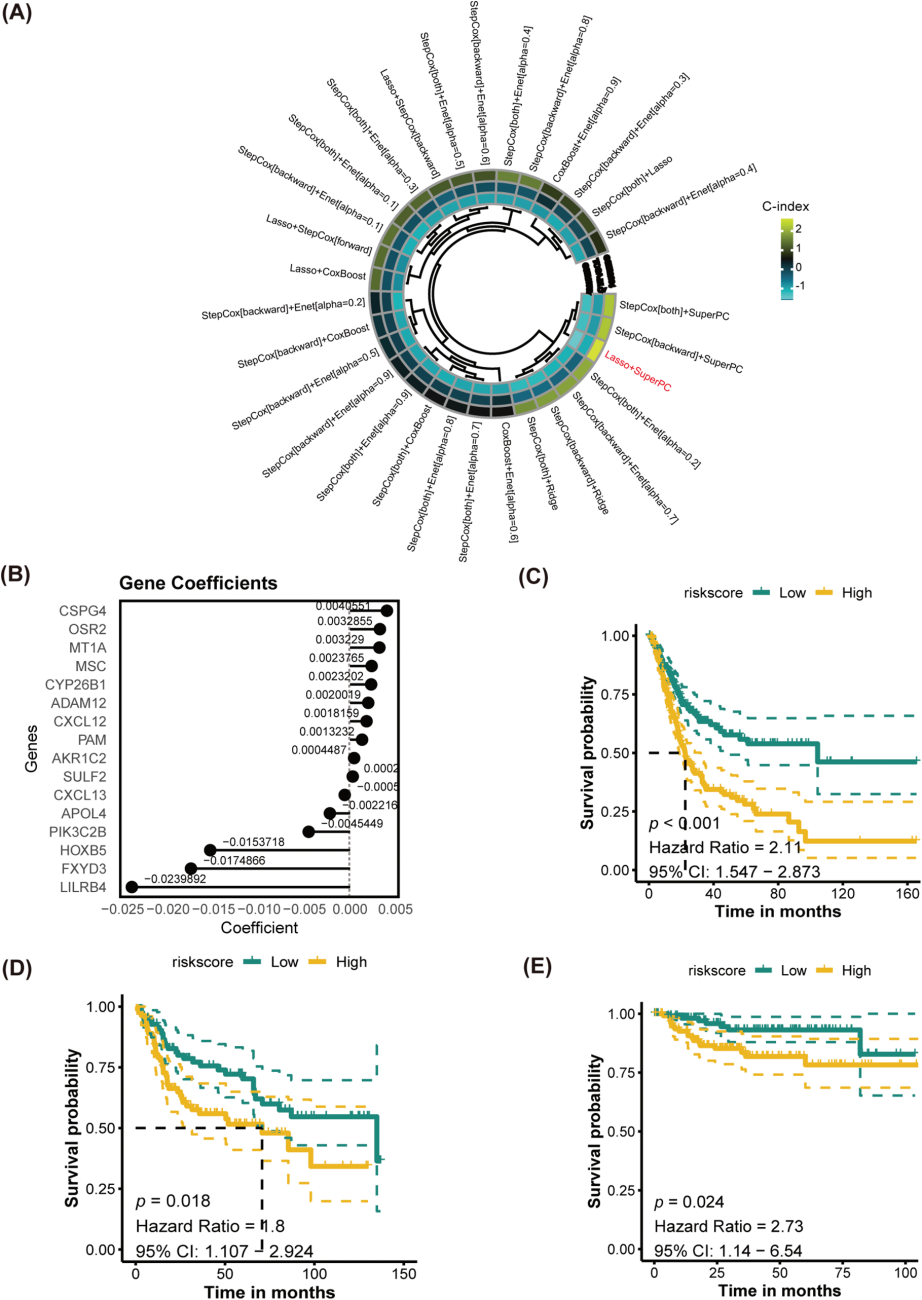
Establishment of PANoptosis-related machine learning prognostic signature (PMLS). **(A)** Average C-index of 30 models established by multiple machine learning algorithms; **(B)** model-established genes and corresponding coefficients; **(C)** K-M analysis for PMLS in TCGA-BLCA cohort; **(D)** K-M analysis for PMLS in GSE32894 cohort; **(E)** K-M analysis for PMLS in GSE13507 cohort

**Figure A5:**
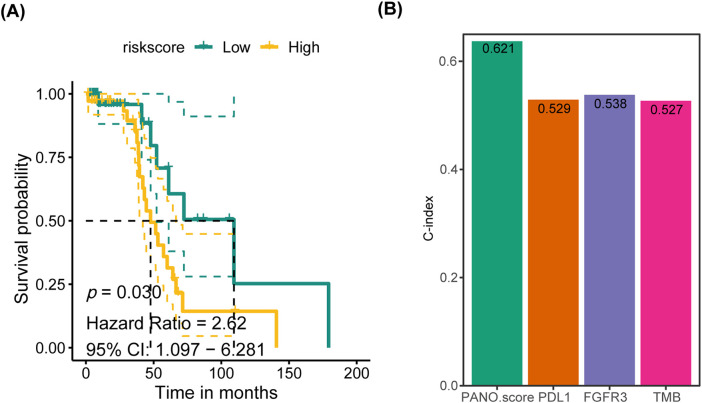
Validation of PMLS in an external cohort. **(A)** K-M plot in GSE48075 cohort; **(B)** the predictive power of PMLS with standard BLCA prognostic biomarkers: PD-L1, FGFR3 and TMB

The training cohort for this study was TCGA-BLCA, while GSE32894, GSE13507 and GSE48075 were used as cohorts for validation. DEGs served as the input for executing a program with 10 classical machine learning algorithms. The average C-index was then computed to determine the most effective algorithm combination. Analysis in Fig. 6A revealed that the highest average C-index was achieved by the LASSO+SuperPC combination. This combination was then utilized to create a machine learning prognostic signature for PANoptosis (PMLS), with the equation: 0.0040551 ∗ CSPG4 + 0.0032855 ∗ OSR2 + 0.003229 ∗ MT1A + 0.0023765 ∗ MSC + 0.0023202 ∗ CYP26B1 + 0.0020019 ∗ ADAM12 + 0.0018159 ∗ CXCL12 + 0.0013232 ∗ PAM + 0.0004487 ∗ AKR1C2 + 0.0002967 ∗ SULF2 − 0.0005864 ∗ CXCL13 − 0.0022166 ∗ APOL4 − 0.0045449 ∗ PIK3C2B − 0.0153718 ∗ HOXB5 − 0.0174866 ∗ FXYD3 − 0.0239892 ∗ LILRB4 (Fig. 6B). PMLS demonstrated a robust predictive capability for OS in BLCA patients, with higher PMLS scores correlating with a worse prognosis. This association held true for TCGA-BLCA (*p* < 0.001, Hazard Ratio = 2.11, 95% CI: 1.547–2.873), GSE32894 (*p* = 0.018, Hazard Ratio = 1.80, 95% CI: 1.107–2.924), and GSE13507 (*p* = 0.024, Hazard Ratio = 2.73, 95% CI: 1.14–6.54) cohorts (Fig. 6C–E). To further validate the performance of the PMLS model, the study utilized the external cohort for independent validation ( Fig. A5). In GSE48075, high PMLS scores showed worse OS in BLCA patients (*p* = 0.030, Hazard Ratio = 2.62, 95% CI: 1.097–6.281). In addition, we compared the discrimination efficiency among PMLS and standard prognostic biomarkers, which include FGFR3, TMB, and PD-L1 expression. The results demonstrated that PMLS presented the highest C-index with 0.621, indicating a favorable predictive power of this novel model (Appendix Fig. A5).

### 3.7 CSPG4 Plays a Pivotal Role in Immunotherapy Efficacy

**Figure 7:**
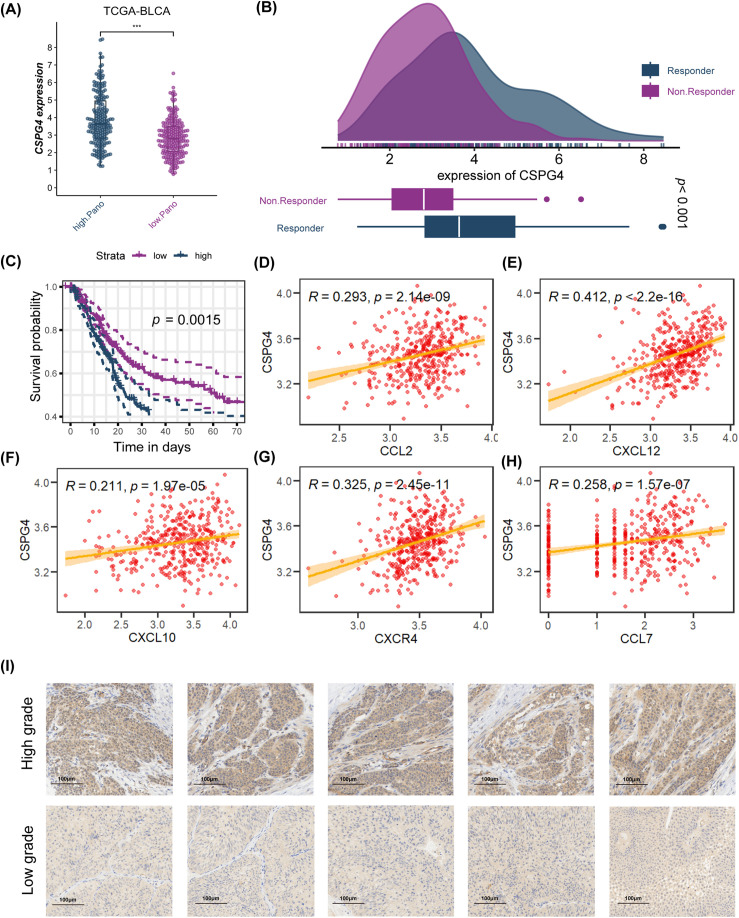
Analysis of CSPG4 in the TCGA-BLCA cohort and immunohistochemistry (IHC) staining of CSPG4 in BLCA patients. **(A)** Comparison of CSPG4 expression between high. PANO and low. PANO subtypes; ***p < 0.001 **(B)** distinct expression of CSPG4 among responder and non-responder patients; **(C)** K-M analysis for CSPG4; **(D)** correlation analysis for CSPG4 and CCL2; **(E)** correlation analysis for CSPG4 and CXCL12; **(F)** correlation analysis for CSPG4 and CXCL10; **(G)** correlation analysis for CSPG4 and CXCR4; **(H)** correlation analysis for CSPG4 and CCL7; **(I)** IHC images illustrating CSPG4 expression in tumor specimens from BLCA patients with either low- or high-grade disease

**Figure A6:**
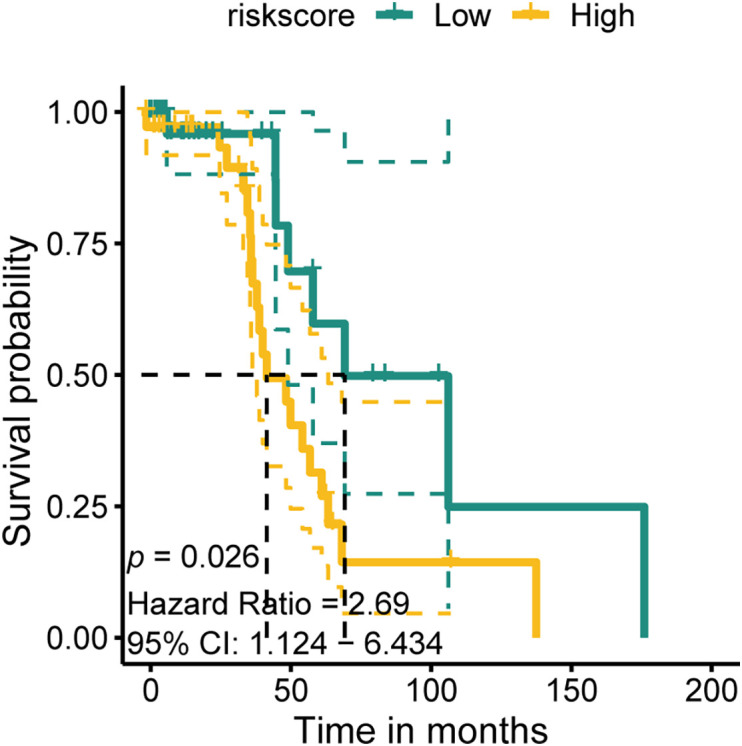
K-M plot in the GSE48075 cohort of CSPG4 for external validation

**Figure A7:**
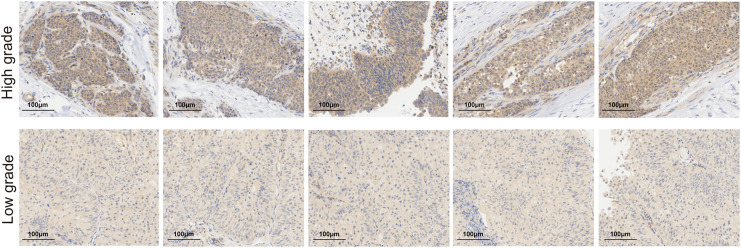
Immunohistochemical (IHC) staining of CSPG4 in tumor tissue from low-grade or high-grade BLCA patients

Based on LASSO and SuperPC analysis, we filtered ten risk genes, where CSPG4 accounts for the most important. we discovered ten risk genes, with CSPG4 emerging as the most significant. Prior research has demonstrated CSPG4 as an indicator of poor prognosis in BLCA and its positive correlation with PD-1 expression, indicating a potential predictive role in immunotherapy response [43]. The study’s analysis unveiled that the high. PANO subtype exhibited notably elevated CSPG4 levels (*p* < 0.001, Fig. 7A). In TIDE analysis, potential TIDE assessment similarly highlighted individuals with the potential to respond well to immunotherapy displayed increased CSPG4 expression (*p* < 0.001, Fig. 7B). High CSPG4 expression displayed worse OS in BLCA patients in TCGA-BLCA (*p* < 0.0015, Fig. 7C) and GSE48075 (*p* = 0.026, Appendix Fig. A6). These results suggest a probable connection between heightened CSPG4 expression, adverse clinical outcomes and a positive immunotherapy response. Additionally, CSPG4 displayed positive associations with CCL2 (R = 0.293, *p* = 2.14e−09), CXCL12 (R = 0.412, *p* < 2.2e−16), CXCL10 (R = 0.211, *p* = 1.97e−05), CXCR4 (R = 0.325, *p* = 2.45e−11), and CCL7 (R = 0.258, *p* = 1.57e−07) (Fig. 7D–H), all of which are identified for their elevated levels in the high. PANO subtype, indicating a potential role of CSPG4 in modulating the inflammatory environment in BLCA. Further IHC of BLCA patients demonstrated that high-grade tumors exhibited heightened CSPG4 expression (Fig. 7I, Appendix Fig. A7), aligning with our cohort analysis.

### 3.8 Novel Nomogram Exhibited Favorable Discrimination Efficiency

**Figure 8:**
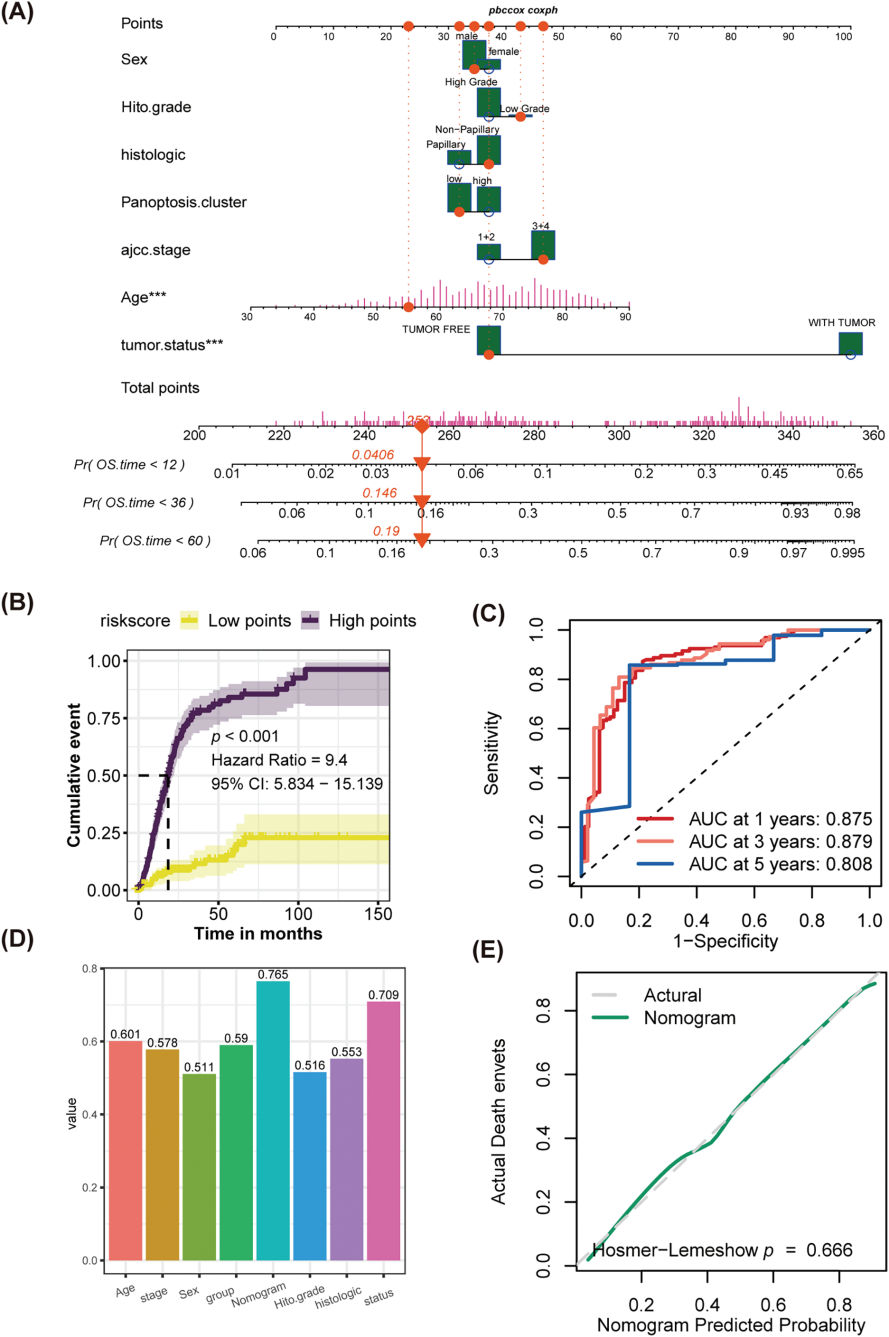
Establishment of a novel nomogram incorporating the PMLS score, and discrimination test and calibration analysis for it. **(A)** The novel established nomogram; ***p < 0.001 **(B)** K-M analysis; **(C)** ROC analysis; **(D)** comparison of C-index among age, stage, sex, PMLS, nomogram, histological grade, histological types, and tumor status. **(E)** Calibration analysis, *p* > 0.05, indicates a good fit between actual events and nomogram-predicted events

Combining multivariate Cox regression analysis and clinical experience, the study filtered independent risk factors to establish a novel nomogram. Employing sex, histological grade, histologic type, AJCC 7th stage, age, tumor status, and PMLS signature as covariates, the study developed a novel nomogram (Fig. 8A and Table 1). The midpoint value of the nomogram scoring system was used to divide patients into two groups, with patients in the high-score category demonstrating significantly worse clinical outcomes than those in the low-score group (Fig. 8B). The area under the curve (AUC) values for 1-year, 3-year, and 5-year were 0.875, 0.879, and 0.808, respectively, indicating robust discrimination efficiency (Fig. 8C). When comparing the C-index among different factors, the nomogram exhibited the highest predictive performance (C-index: 0.765) (Fig. 8D). Calibration analysis manifested that the predicted probabilities were in close fitness of the actual outcomes, showing a high congruence between predicted and observed events (Hosmer-Lemeshow, *p* = 0.666) (Fig. 8E).

**Table 1:** Multivariate Cox regression analysis

		N	HR	95% Cl	*p*
**Age**	–	348	1.030	1.012~1.048	0.001
**Sex**	Female	87	–	–	–
	Male	261	0.970	0.671~1.402	0.873
**Group**	High	165	–	–	–
	Low	183	0.627	0.442~0.891	0.009
**Tumor status**	With tumor	152	–	–	–
	Tumor free	196	0.139	0.091~0.213	<0.001
**AJCC stage**	1 + 2	120	–	–	–
	3 + 4	228	1.438	0.946~2.185	0.089
**Histological grade**	High-grade	–	–	–	–
	Low-grade	20	1.264	0.293~5.442	0.753
**Histological type**	Papillary	113	–	–	–
	Non-papillary	235	1.110	0.733~1.682	0.623

Note: Events, 143; Global *p*-value (Log-Rank), 2:4038e−29; AIC, 1348.02; Concordance Index, 0.77; AJCC, American Joint Committee on Cancer.

## 4 Discussion

PANoptosis is a distinct form of PCD that incorporates pyroptosis, apoptosis and necroptosis. This phenomenon has garnered interest in cancer research due to its possible influence on tumor immunity, cancer prognosis, and the effectiveness of immunotherapy. In the context of bladder cancer, investigations into PANoptosis are still in the early phases, with researchers exploring its potential as both a therapeutic target and a prognostic indicator [44].

The prognostic impact of PANoptosis in cancer remains a contentious issue. Some views argue that it can enhance tumor eradication by promoting tumor cell death, potentially improving therapeutic efficacy. Conversely, certain forms of PANoptosis might enable tumor cells to evade treatment through the activation of anti-apoptotic pathways [45–47]. This research examines the role of PANoptosis in BLCA, with a particular focus on its implications for prognosis and immunotherapeutic interventions. The study identified two distinct patterns of PANoptosis within BLCA, each correlating with unique clinical endpoints and treatment responses. The high. PANO subtype was associated with reduced overall survival and a greater tendency to develop advanced tumors. Additionally, this subtype exhibited heightened activation of cell-cycle-relevant signaling pathways, implying a rapid proliferation rate. Notably, high. PANO also demonstrated elevated scores for PYROPTOSIS, APOPTOSIS, and NECROPTOSIS, indicating possible interactions among different programming death pathways and reinforcing the idea of a unified death network [29].

PANoptosis is a type of PCD characterized by inflammation that occurs in both infections and autoinflammatory diseases [12]. In inflammatory tumors exhibiting elevated levels of PANoptosis, there is typically a significant rise in the presence of immune cells like dendritic cells, CD8^+^ T cells, and macrophages [48]. These cells are essential to anti-tumor responses, particularly in the context of immune checkpoint inhibitor therapy, as they facilitate antigen presentation and the elimination of tumor cells [49]. While a heightened state of PANoptosis may lead to the buildup of Tregs and cancer-associated fibroblasts (CAFs) within the tumor microenvironment, these components are often suppressed by immune checkpoint inhibitors, thereby mitigating their negative impact on immunotherapy outcomes [44,50,51]. Several research studies have indicated that a high degree of PANoptosis is indicative of a favorable response to immunotherapy. For example, Ouyang and colleagues discovered that individuals exhibiting high levels of PANoptosis-associated genes were likely to respond positively to immunotherapy in cases of hepatocellular carcinoma [21]. Similarly, in instances of clear cell renal cell carcinoma, a pronounced PANoptosis trait was linked with elevated invasion of immune cells, promoted level of immune checkpoint expression, and the potential to derive benefits from immunotherapy [52]. This study’s findings additionally revealed enhanced activation of immune-related pathways, including inflammatory responses and immune cell infiltration, within the subset showing high PANoptosis, suggesting a potential receptiveness to immunotherapy. This was further corroborated by analyses conducted using TIDE and SubMap methods.

The prognostic signature linked to poor outcomes in BLCA was further validated using machine learning algorithms. A key marker, CSPG4, was identified to correlate with unfavorable prognosis and increased effectiveness of immunotherapy. Also referred to as NG2 or Chondroitin Sulfate Proteoglycan 4, CSPG4 is a cell surface protein with significant roles in various biological processes and diseases, including cancer [53–55]. In the context of malignant disease, CSPG4 expression might be an essential part in metastasis and progression, making it a latent therapeutic target. While few studies have investigated CSPG4 in BLCA, it is potentially upregulated in tumor tissues. This upregulation has been linked to cancer progression and may reflect unfavorable clinical outcomes [55]. High. PANO tumor also had higher expression of CSPG4, and K-M analysis validated that high-expression tumors had shorter OS in BLCA [39,56]. Importantly, potential responders to immunotherapy also highly expressed CSPG4, indicating it might be an indicator for immunotherapy in BLCA.

Nevertheless, this research does have some restrictions. Although three outside cohorts were verified, the total size of the sample is still restricted and might not be enough to entirely depict the condition of all individuals with BLCA. The selection of samples might be biased, given that the utilization of public series datasets could be limited by geography and population and other factors. TCGA and GEO datasets may be biased toward specific ethnic groups and tumor subtypes, affecting the generalizability of findings. Future studies should include diverse and multicenter cohorts for broader applicability. The study preliminarily explored the role of CSPG4 in triggering the PANoptosis program and promoting the tumor progression of BLCA, and further validation based on experiments is required, which would be performed in future work.

## 5 Conclusion

This study identified two distinct patterns of PANoptosis in BLCA using cluster analysis. The high-PANO subtype exhibited significantly elevated cell death pathway activation scores and was linked to worse overall survival and higher tumor stage. Despite poor prognostic indicators, there may be a better response to immunotherapy, possibly influenced by CSPG4. Furthermore, a new nomogram with excellent predictive performance was developed.

## Data Availability

The data of this study are available in the TCGA dataset (https://tcga-data.nci.nih.gov/tcga/) and GEO (https://www.ncbi.nlm.nih.gov/gds (accessed on 18 May 2025)).

## Abbreviations

BLCA: Bladder cancer
TIDE: Tumor immune dysfunction and exclusion
High. PANO: High PANoptosis
Low. PANO: Low PANoptosis
PD-1: Programed cell death protein-1
PPI: Protein-protein interaction
OS: Overall survival
PMLS: PANoptosis-related machine learning prognostic signature
PCD: Programmed cell death
RIPK1: Receptor-interacting protein kinase 1
RIPK3: Receptor-interacting protein kinase 3
PRRs: Pattern recognition receptors
PAMPs: Pathogen-associated molecular patterns
DAMPs: Damage-associated molecular patterns
TCGA: The Cancer Genome Atlas
GEO: Gene Expression Omnibus
TPM: Transcripts per million
KEGG: Kyoto Encyclopedia of Genes and Genomes
TMB: Tumor mutational burden
CDF: Cumulative distribution function
GSVA: Gene Set Variation Analysis
DEGs: Differentially expressed genes
GO: Gene Ontology
GSEA: Gene Set Enrichment Analysis
NES: Normalized enrichment score
FDR: False discovery rate
TIDE: Tumor immune dysfunction and exclusion
IHC: Immunohistochemistry
FFPE: Formalin-fixed paraffin-embedded
DAB: Diaminobenzidine
AJCC: American Joint Committee on Cancer
K-M: Kaplan-Meier
ROC: Receiver operating characteristic
RISC: RNA-induced silencing complex
Tregs: Regulatory T cells
MDSCs: Myeloid-derived suppressor cells
AUC: Area under the curve
CAFs: Cancer-associated fibroblasts
